# Dengue outbreak and fragile healthcare system: Doctors at the verge of mental and physical stress

**DOI:** 10.1002/brb3.2708

**Published:** 2022-09-27

**Authors:** Syeda Tayyaba Rehan, Muhammad Sohaib Asghar, Irfan Ullah, Hamid Mahmood, Ka Yiu Lee, Muhammad Junaid Tahir

**Affiliations:** ^1^ Department of Internal Medicine, Dow University of Health Sciences Karachi Pakistan; ^2^ Kabir Medical College Gandhara University Peshawar Pakistan; ^3^ Department of Internal Medicine, Lahore General Hospital Lahore Pakistan; ^4^ Swedish Winter Sports Research Centre, Department of Health Sciences Mid Sweden University Östersund Sweden

**Keywords:** COVID 19, dengue, healthcare workers, mental health, Psychiatry

1

Dengue fever is one of the most prominent emerging arboviral infections and serves as a leading cause of death in Asian and Latin American countries. According to the World Health Organization (WHO), the burden of dengue fever has grown dramatically over the last two decades, with half of the world's population currently at risk of this lethal infection. (WHO, 2021). The WHO, on May 19, 2021, declared this *Aedes* mosquito‐laden infection endemic in approximately 100 countries (WHO, 2021).

As documented by Bhatt et al., there are approximately 400 million cases of dengue fever recorded each year, of which 96 million patients need clinical management of the disease every year (Bhatt et al., 2013). From 2000 to 2015, deaths from dengue increased from 960 to 4032 (WHO, 2021). The burden of infection is concentrated up to 70% in developing Asian countries, specifically those struggling with inadequate healthcare systems (Bhatt et al., 2013).

When the coronavirus disease 2019 (COVID‐19) pandemic is already putting immense pressure on healthcare staff and affecting their physical and mental health (Ho et al., 2020), a dengue outbreak is discerning the further collapse of the fragile healthcare system. The co‐occurrence of COVID‐19 and dengue fever served as a major drawback for Asian countries having inadequately facilitated hospitals. As reported on October 6, 2021, the isolated wards established in Pakistan for dengue patients in major hospitals of the city went out of the space for critical patients visiting with low platelets (The Dawn Newspaper, 2021). Misdiagnosis of COVID‐19 infection as dengue fever was also noticed by several surveys conducted globally (Harapan et al., 2021). This could be due to the serological cross reactivity of severe acute respiratory syndrome coronavirus 2 antigen and Dengue virus antibodies or due to the similar clinical features of both infections (Harapan et al., 2021). Studies have reported that 25% of patients with confirmed dengue diagnoses share symptoms of COVID‐19, such as cough and upper respiratory tract symptoms. Similarly, COVID‐19 may manifest itself as fever with muscle and joint pain without respiratory symptoms, thus mimicking dengue infection, especially in infants. Patients with these symptoms must be investigated for both diseases to make a confirmed diagnosis (Nacher et al., 2020). Delayed diagnosis and later management of COVID‐19 infection can further overwhelm the ongoing rush of critical patients within hospitals and push frontline workers to increased duty hours and momentous responsibilities.

A study by Ehelepola and Wijesinghe observed that disease outbreaks carry a high potential to adversely affect frontline healthcare workers (HCWs) in various aspects (Ehelepola & Wijesinghe, 2018). The overwhelming influx of patients, responsibility of on‐time critical decisions, outweighing potential serious consequences, and pressure to avoid medical errors were among a few variables confronted by HCWs that rendered the medical practice inherently stressful (Boo et al., 2018). A multicentric cross‐sectional survey conducted in major Malaysian hospitals documented that 41.5% of HCWs serving dengue wards experienced high emotional exhaustion, 35.5% of the survey respondents showed high depersonalization, and 61.3% of respondents reported low personal accomplishment (Boo et al., 2018). The authors of this survey further stated that 15.9% of the doctors were experiencing severe burnout symptoms. A systematic review by Chirico et al. disclosed that during the COVID‐19 pandemic, 7.4%–37.4% of HCWs suffered from posttraumatic stress disorder. In Asian countries, the fear rate among Asian HCWs was recorded as 77.1% (Chirico et al., 2021).

Noticeable factors that preceded the HCWs toward burnout symptoms included increased working hours, escalating depression, anxiety, and stress during the outbreak (Boo et al., 2018). Media highlights of the dengue fever and negative portrayal of the HCWs reported to escalate anxiety among HCWs during the dengue outbreak (Ehelepola & Wijesinghe, 2018). Some of the factors that can root anxiety and stress among doctors during the COVID‐19 pandemic include the lack of space in hospitals for critical patients and an inadequate supply of equipment (Alnazly et al., 2021).

Increased working hours, altered sleep patterns, and risk of infection contraction were reported as major problems causing lack of motivation and avoidance behavior among HCWs during different outbreaks (Stewart et al., 2021). The stress of providing optimal care for patients with an inadequate supply of equipment and investigatory facilities has been identified as a potential factor that may lead to negative health effects such as burnout in medical doctors (Williams et al., 1997).

Several factors can escalate mental and physical stress among doctors during dengue outbreaks. Compromised mental health might hinder the quality functioning of HCWs during such outbreaks. The inability of frontline workers to deliver quality work can further demolish the fragile healthcare system. A better understanding of all the intrinsic and extrinsic factors producing adverse effects on medical professionals’ health is urgently needed. Furthermore, screening of depression, stress, and burnout symptoms in doctors must progress at an early stage to curb the risk of resulting complications. The government should channel efforts in providing psychological first aid facilities to HCWs, and regular surveys must be conducted to assess the fear‐elevating risk factors among HCWs. Expert psychologists must be taken on board to conduct facultative workplace health promotion programs. Preventive interventions should be induced in a timely manner for HCWs by training them in evidence‐based anticipatory methods of coping with stressful events. Increasing awareness about the outbreak among the general population, hiring more doctors and paramedical staff in hospitals, providing extra incentives, and taking help from telehealth services could be beneficial.

## CONFLICTS OF INTEREST

The authors declare no conflict of interest.

## AUTHOR CONTRIBUTIONS

Muhammad Junaid Tahir, Syeda Tayyaba Rehan, and Irfan Ullah conceived the idea. Syeda Tayyaba Rehan, Muhammad Junaid Tahir, Muhammad Sohaib Asghar, and Irfan Ullah performed a literature review and wrote the initial manuscript. Hamid Mahmood, Muhammad Sohaib Asghar and Ka Yiu Lee reviewed the manuscript and critically revised it to the final form. All authors approved the final version of the manuscript.

### PEER REVIEW

The peer review history for this article is available at: https://publons.com/publon/10.1002/brb3.2708.

## Data Availability

Data are available from the first author upon reasonable request.
